# Clinically Relevant Post-Translational Modification Analyses—Maturing Workflows and Bioinformatics Tools

**DOI:** 10.3390/ijms20010016

**Published:** 2018-12-20

**Authors:** Dana Pascovici, Jemma X. Wu, Matthew J. McKay, Chitra Joseph, Zainab Noor, Karthik Kamath, Yunqi Wu, Shoba Ranganathan, Vivek Gupta, Mehdi Mirzaei

**Affiliations:** 1 Department of Molecular Sciences, Macquarie University, Sydney, NSW 2109, Australia; dana.pascovici@mq.edu.au (D.P.); jemma.wu@mq.edu.au (J.X.W.); matthew.mckay@mq.edu.au (M.J.M.); zainab.noor@students.mq.edu.au (Z.N.); Karthik.kamath@mq.edu.au (K.K.); Yunqi.wu@mq.edu.au (Y.W.); Shoba.ranganathan@mq.edu.au (S.R.); 2 Australian Proteome Analysis Facility, Macquarie University, Sydney, NSW 2109, Australia; 3 Department of Clinical Medicine, Macquarie University, Sydney, NSW 2109, Australia; chitra.joseph@hdr.mq.edu.au (C.J.); vivek.gupta@mq.edu.au (V.G.)

**Keywords:** post translational modification, quantitative proteomics, body fluids, clinical samples, PTM

## Abstract

Post-translational modifications (PTMs) can occur soon after translation or at any stage in the lifecycle of a given protein, and they may help regulate protein folding, stability, cellular localisation, activity, or the interactions proteins have with other proteins or biomolecular species. PTMs are crucial to our functional understanding of biology, and new quantitative mass spectrometry (MS) and bioinformatics workflows are maturing both in labelled multiplexed and label-free techniques, offering increasing coverage and new opportunities to study human health and disease. Techniques such as Data Independent Acquisition (DIA) are emerging as promising approaches due to their re-mining capability. Many bioinformatics tools have been developed to support the analysis of PTMs by mass spectrometry, from prediction and identifying PTM site assignment, open searches enabling better mining of unassigned mass spectra—many of which likely harbour PTMs—through to understanding PTM associations and interactions. The remaining challenge lies in extracting functional information from clinically relevant PTM studies. This review focuses on canvassing the options and progress of PTM analysis for large quantitative studies, from choosing the platform, through to data analysis, with an emphasis on clinically relevant samples such as plasma and other body fluids, and well-established tools and options for data interpretation.

## 1. Introduction

The ability to analyse protein post-translational modifications (PTMs) occurring on a large scale in a biological system yields insight into their roles and relevance to disease states and confers proteomics a unique edge. An immense variety of biological responses occur in this manner—in Bill Bryson’s memorable turn of phrase “depending on mood and metabolic circumstance, (proteins) will allow themselves to be phosphorylated, glycosylated, acetylated, ubiquitinated, farnesylated, sulphated and linked to GPI anchors, among rather a lot else” [1]. The wealth of these changes and their importance in cell signalling and disease led to modified proteins being the focus of clinical and pharmaceutical research as potential drug targets [2,3,4]. Several factors have converged to make the analysis of such modifications possible on a large scale: advances in mass spectrometry (MS) methods including larger multiplexing chemical labelling (Isobaric tag for relative and absolute quantitation—iTRAQ, Tandem Mas Tag—TMT) and other novel label-free quantitation approaches such as DIA/SWATH [5,6], improvements in PTM enrichment strategies [7], the development of more detailed and robust PTM workflows [8,9], and crucially, improvement in bioinformatics tools and databases making the subsequent analysis possible. In generic terms, the ideal goals of all such large scale PTM analyses are easy enough to state: canvass which sites on the proteins are modified, quantify how those modifications change with condition or disease, and determine what the modification achieves in terms of function in the cell.

**Figure 1:**
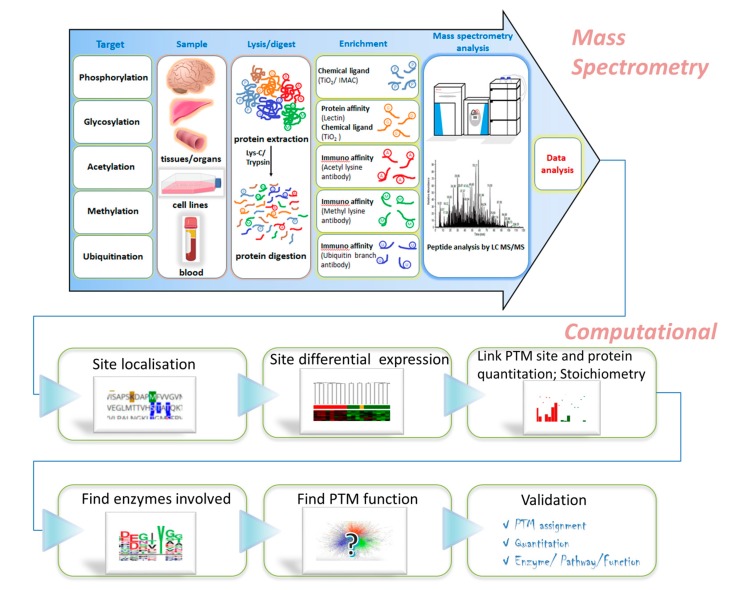
Schematic PTM Workflow illustrating some of the common steps associated with sample preparation, PTM enrichment, MS and bioinformatics analysis.

Recent reviews illustrated the potential of PTMs as disease biomarkers [10], surveyed disease associated PTM changes [11], in cardiovascular disease [12], cancer [13], neurodegenerative disease [14] and diabetes [15], demonstrating a growing interest in characterising PTMs to answer clinical questions [16]. The present review is structured to follow the workflows necessary for undertaking a large scale PTM experiment aiming at the ideal goals described above, in logical order from the sample preparation through to the data analysis, with emphasis on the bioinformatics steps needed. Figure 1 captures the workflow and outlines some of the common approaches used in PTM studies. Our focus is on studies on human plasma and other body fluids and clinically relevant cell lines, and for the sake of clarity we limit our consideration to the five modifications whose study has matured the most: phosphorylation, glycosylation, acetylation, methylation and ubiquitination. The first two sections provide the background in describing the modifications surveyed and their clinical relevance, giving known examples of such studies in relevant body fluids. Section 3 and Section 4 discuss the quantitative mass spectrometry methods amenable to large scale PTM studies, and review the optimum enrichment methods, including some practical aspects and challenges. The remaining sections focus on the bioinformatics and statistical analysis aspects, structured into four basic areas: site localisation (understand which sites are modified), assessments of quantitation and stoichiometry (how quantitation changes with condition or disease and what proportion of peptides is modified), through to available tools for the subsequent analysis and finally network visualisation approaches as well as validation analysis (assign and validate function).

## 2. Modifications with Known Clinical Relevance

**Figure 2:**
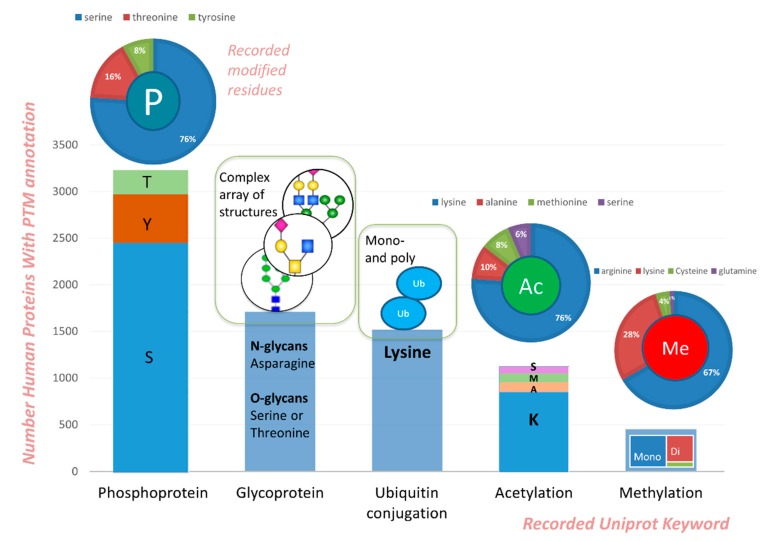
Human proteins with PTM currently available in the Uniprot database; percentage of modified residues is indicated above the bars, wherever that information is available.

The complex phenotype of an organism is attributable to the extensive and diverse phenomenon of protein regulation; this hidden regulation of the protein network is made possible by post translational modifications. A single PTM can re-establish the entire downstream trafficking transforming the protein function and cell fate. Hence PTMs determine the optimal functionalities of several proteins that are involved in an array of physiological and disease states and as such comprises the basis of several drug targeting strategies and diagnostic tests. PTMs determine the protein interactions with other proteins and form the basis of several cellular signalling pathways. These also regulate the cell–cell and cell–matrix interactions and play vital roles in inflammation, host-pathogen interactions, immune modulation, and degenerative and proliferative disorders. According to reports, 100 of the 469 existing PTMs in the UniProt database are found in humans [16]. Although several PTMs have been found to date, transient modifications and low abundances account for some of the many challenges that limit our understanding of PTMs. Here we highlight some examples of known PTMs reported to have clinical relevance in disease pathology and which have considerable information amassed about them in public databases (Figure 2).

Phosphorylation is one of the most widely studied modifications and can occur in a very dynamic and rapid manner, regulating various signalling pathways in health and disease conditions. The PhosphoSite Plus database records more than 350 proteins with modified PTMs under disease conditions. Phosphopathology is a long-debated concern in neurodegenerative disorders. Early records of hyperphosphorylated neurofibrillary tangles (NFT) in Alzheimer affected brains have been reported as long ago as the 1980s [17,18]. Tau hyperphosphorylation, the major constituent of NFT is another example of disrupted PTM in Alzheimer’s disease (AD) [19]. Detection of Tau phosphorylation in cerebrospinal fluid (CSF) might reflect AD progression and suggest pathological mechanisms underlying the disease [20]. Phosphorylation at S129 of α-synuclein is associated with synucleinopathy lesions like Parkinson’s disease and dementia [21,22]. Atypical phosphorylation patterns of specific proteins are observed in several malignancies as evidenced in non-small cell lung cancer (NSCLC) patient tumour samples [23], serum samples from breast and prostate cancer patients [24], patient-derived acute myeloid leukaemia bone marrow cells (AML) [25], human pancreatic duct tissue of Pancreatic ductal adenocarcinoma patients [26] and renal cell carcinoma tumours from kidney cancer patients [27]. Cardiovascular diseases are also influenced by erroneous protein phosphorylation. A detailed review by Rapundalo et al. has addressed the essential proteins that are negatively modulated by phosphorylation leading to cardiac dysfunction [28].

Glycosylation is another common PTM, which plays an important role in a protein’s structure and function and has been shown to be involved in evolution, development and immunity. Glycoproteomics—the characterisation of the oligosaccharides (glycans) attached to proteins—is an important challenge. Glycans are a chemically diverse structural feature of proteins originating from the enzymatic addition of individual monosaccharide units to asparagine (*N*-glycans) or serine and/or threonine (*O*-monosaccharides and *O*-glycans). The result is a complex array of monosaccharides or glycan structures with different compositions, lengths and linkages.Glycoproteomics is a rapidly emerging field and several modifications and their relevance have been elucidated in recent years. Valero et al. suggested glycosylation of proteins acetylcholinesterase and butyrylcholinesterase in AD patient antemortem tissues as markers of disease progression [29,30,31]. Creutzfeldt-Jakob disease (CJD) is another example where the pathological glycosylation profile of CSF acetylcholinesterase enzyme has been identified [32]. Specific glycosylation patterns of proteins in various brain regions such as the frontal cortex in Parkinson’s disease and temporal lobe samples in Amyotropic Lateral Sclerosis (ALS) have also been reported [33,34]. In breast cancer, glycan changes were shown to mediate disease progression and influence overall survival rates [35]. Supporting these studies, the mRNA and protein levels of core 1 β-1,3-galactosyltransferase (C1GALT1) were observed to be elevated in hepatocellular carcinoma [36]. Similarly, detailed studies reveal genes modifying glycosylation patterns contribute towards disease pathology in different cancer types [10,11,12,13]. Glycated haemoglobin measurement is an important diagnostic test for blood sugar levels over an extended period of time in diabetes [37].

Ubiquitin (Ub) is a small protein composed of 76 amino acids, which can covalently modify other proteins, usually at lysine residues. Ubiquitin is a highly conserved protein often involved in protein degradation, and is implicated in several pathophysiological states including cancer and neurodegeneration. Ubiquitination is a reversible process which involves cleavage of ubiquitin by deubiquitin enzymes (DUBs). Poly ubiquitination is known to target proteins for degradation via 26S proteasome and to recycle ubiquitin. The modification occurs between the C-terminus of Ubiquitin and the amino group of lysine residues, or sometimes the N-terminus of the substrate protein. Ubiquitination can involve mono- or poly ubiquitination and each of the seven lysine residues (Lys-6, Lys-11, Lys-27, Lys-29, Lys-33, Lys-48, and Lys-63) in Ubiquitin itself can serve as potential modification sites for continued poly ubiquitination.

Severe ubiquitin overexpression is observed in lung cancer tissues and a recent study demonstrated the role of E3 ubiquitin ligase NEDD4 in enhancing the EGFR mediated migratory potential of NSCLC cells [38,39]. E3 ubiquitin ligase WWP1 was detected in high levels in bone marrow tissue in acute myeloid leukaemia [40]. Ubiquitin protein ligase E3C (UBE3C) was upregulated in renal cell carcinoma tissues and was reported to be associated with poor survival rate [41]. SPOP mediated ATF2 ubiquitination has been reported in prostate cancer samples [42]. Ubiquitin-conjugating enzyme Ubc13 was found to be upregulated in malignant tissues from breast, pancreas, colon, prostate, and lymphoma [43]. Neurodegenerative disorders are also not exempt from the deleterious effects of ubiquitination as illustrated by the following clinical scenarios. Perry et al have demonstrated the co-existence of ubiquitin in Neurofibrillary tangles (NFT) and neurites associated with senile plaques in AD brain tissues [44]. Lewy bodies in Parkinson’s disease show immunoreactivity to ubiquitin providing insights into the shared PTM feature of several neurodegenerative conditions [45]. Dysregulated ubiquitination was also reported to underlie cardiotoxicity in dilated cardiomyopathy (DCM) and cardiac explants from DCM patients showed hyperubiquitination of proteins with increased mRNA levels of proteins involved in ubiquitin-proteosome pathway [46]. A detailed review on the role of ubiquitination in innate and adaptive immunity highlights the potential implications ubiquitination has on autoimmune disorders as well [47].

Acetylation and methylation are epigenetically relevant PTMs that have well-established roles in disease phenotype by altering replication and transcription regulation. Methylation of arginine by protein methyl transferases (PRMTs) is known to regulate transcription, cell signalling and cell fate. The addition of methyl groups is via covalent attachment to the side chain of arginine and can be either monomethylarginine (monoisotopic, 14.0157 Da), dimethylarginine (symmetric or asymmetric, monoisotopic, 28.0313 Da) or trimethylarginine (monoisotopic 42.0470 Da). Acetylation of the N-terminus (NTA or *N*-acetylome) by N-terminal acetyltransferases (NATs) plays an important role in protein structure and function. The reversible acetylation of lysine (K-acetylome) by lysine acetyltransferases (KATs) and histone deacetylases (HDACs) plays an important role in histone modification and the regulation gene expression, but the functional changes of reversible acetylation are likely to be far broader than the histones and gene regulation.

Differential expression in the methylation of histone PTM sites of human AD frontal cortex samples contributed towards the disease pathology [48]. Similarly, an increased histone methylation profile was observed in monocytes from type 1 diabetes patients, and a parallel increase in the acetylation pattern was also detectable in these patient samples [49]. Genome-wide expression studies revealed elevated protein arginine methyl transferases 1 and 6 (PRMT1 and PRMT6) expression in lung, pancreatic, breast, and urinary bladder cancers [50,51,52,53]. Chronic obstructive pulmonary disease (COPD) marked the expression of hyperacetylated histone h3.3 rendering resistance to UPS mediated degradation in the airway lumen, alveolar fluid, and plasma samples collected from COPD patients [54]. A significant increase in histone deacetylase 6 levels was evident in oral squamous cell carcinoma with a correlation to tumor aggressiveness suggesting the key roles played by protein deactylation in biochemical processes [55]. Tampered HDAC1 and class II HDAC regulation were evident in human prostate cancer and lung cancer specimens [56,57]. Additionally, rogue acetylation associated with mutant p53 proteins was observed in several tumors [58]. Histone deacetylase inhibitors have been shown to provide neuroprotection to the retinal ganglion cells in experimental model of optic nerve injury [59].

## 3. Examples of PTM Studies in Plasma or Other Clinically Relevant Fluids

With the rapid advancement in mass spectrometry techniques researchers can gain detail of relevant PTMs in multiple patient samples. Owing to the role played by PTMs in disease, techniques that can analyse PTMs in patient derived biological fluids open up a new horizon for biomarker discovery research. Plasma, serum and other biological fluids are attractive clinical samples for the purpose of discovering disease-relevant biomarkers. The advantage of using such biological fluids for PTM analysis lies in the ease of accessibility and the promise of developing personalised medicine approaches better suited to each individual.

Table 1 highlights several studies carried out in various biological fluids with findings related to the impact of post-translational modifications on disease. The most commonly used samples, not surprisingly, remain plasma and serum. Urine and saliva are other easily accessible samples of general interest; however, information is still lacking on disease-relevant urine or saliva PTMs. More specific body fluids such as cerebrospinal fluid are naturally of high interest for neurological disease studies.

In terms of specific PTMs, protein glycosylation and phosphorylation are the most widely reported PTMs recognised in body fluids relevant to pathological. Aberrant glycation identified in patient serum samples of multiple cancer types indicate the potential of glyco bio-markers in tumor diagnosis, while circulating phosphorylated proteins have been reported extensively in neurodegenerative disorders.

## 4. Quantitative MS Methods for PTMs Analysis

Mass spectrometry techniques are capable of assessing the modification status of proteins including site localisation and occupancy [84,85,86]. Workflows for PTM analysis often focus on a single modification type—phosphorylation or glycosylation, etc.—and one sample type (blood, plasma, tissue, or cell lines). Substantial progress in PTM analysis has come from cell lines studies, providing the foundations for developing mature PTM workflows, including the establishment of protocols for sample preparation, strategies for PTM enrichment, and sensitive LC-MS/MS detection for PTM quantification. These workflows often make use of carefully selected lysis and digestion protocols, selective purification techniques based on affinity or immunoprecipitation enrichment and quantitative mass spectrometry. It is worth mentioning that these protocols are generally achieved using larger amounts of starting materials (mgs of proteins/peptides) not necessarily ideal for the clinical samples with lower amount of proteins (CSF, tear or saliva). The following section highlights common workflows used for the analysis of phosphorylation, glycosylation, acetylation, methylation, and ubiquitination using quantitative mass spectrometry.

A detailed description of MS methodology including chromatography and mass spectrometry instrumentation is included in the recent phosphoproteomics review of Riley and Coon [87], while data acquisition methods were very recently and comprehensively reviewed in the context of PTM analysis [12], and details on sequence-specific identifications of PTMs including example spectra are provided in the classic review of Choudhary and Mann [88].

### 4.1. MS General Considerations: Sample Collection And Digestion

Although mass spectrometry has been used extensively to identify proteins and their modifications, large-scale proteome expression profiling in combination with comprehensive PTM site localisation and quantification is a rather ambitious challenge, particularly in the context of spatial and temporal profiling. For clinically relevant studies, stringent SOPs addressing sample collection are required and there is often a need to include reagents and enzymes to inhibit sample degradation after collection and before analysis [10]. For instance, for plasma proteomic studies, it is common practice to use blood collection tubes containing additives, or to introduce inhibitors to avoid undesirable endogenous enzyme activity. As an example, commercially available phosphatase inhibitors are used to avoid undesirable activities of phosphatase enzymes during enrichment of phosphopeptides.

Quantitative assessment of PTMs in the context of clinically relevant studies, represent a very important stepping stone on the path to understand human pathologies. Many quantitative LC-MS/MS approaches make use of the “bottom-up” proteomics strategy, which involves the digestion of proteins using a proteolytic enzyme such as trypsin (or a combination of endopeptidases such as Lys-C and trypsin), then LC-MS/MS analysis and identification of peptides using a protein sequence database and search algorithm. This approach is well suited to analysing PTMs, particularly for the smaller modifications (e.g., phosphorylation, methylation and acetylation), and is now used to identify and quantify different modified and unmodified proteoforms in parallel. Many smaller PTMs introduce a predictable mass shift which can be used to identify the site of modification. However, for larger modifications such as glycosylation, the path to identifying the site and composition of glycans attached to glycoproteins is more challenging, and database search algorithms are not particularly useful in most instances. Instead, various approaches using chemical labelling and cleavage of the glycans are used to identify the site of modification, and more importantly the structure of released glycans.

A major limitation of the bottom-up approach is introduced at the digestion step—the proteoform-origin of each individual peptidoform is lost. For modified peptides, each specific modification site represents a unique peptidoform to be quantified. However, in the context of expression proteomics, unmodified peptides are usually grouped together and differential analysis is used to determine the significance of any change in expression attributed to a change in sample conditions. This approach confounds the parallel quantification of individual peptidoforms arising from a modification and disconnects it from the protein level quantification of each protein/proteoform. It also masks accurate quantification of individual proteoforms arising from sequence mutations or splice variants. Peptide level quantification may yet play an important role in LC-MS/MS-based proteomics.

### 4.2. MS General Considerations: Stochastic Nature of Acquisition

The MS techniques used for many large-scale proteomic studies rely on Data Dependent Acquisition (DDA). Although modern MS instrumentation allows for increasingly fast data acquisition rates, providing ever increasing numbers of peptide-spectrum matches (PSMs), DDA is stochastic in nature, and missing values in individual DDA experiments lead to a degree of incompleteness in large data sets. Data Independent Acquisition (DIA) approaches such as the Sequential Windowed Acquisition of All Theoretical Fragment Ion Mass Spectra (SWATH-MS) can go some way to overcoming missing values in quantitative studies; however the libraries used to generate reference spectra for identification purposes are currently almost exclusively derived from DDA experiments—so the stochastic element of DDA still remains a challenge. Pooling samples from different groups, treatments or conditions in combination with sample fractionation such as high pH chromatography, can reduces the limitations of stochastic DDA experiments, and this approach has been used to successfully quantify PTMs in SWATH-MS experiments—SWATHProphetPTM [89]. To address missing values and the need for imputation, the inference of peptidoforms (IPF) approach, has been used to target the detection of phosphopetides and multiple PTM peptidoforms in plasma, demonstrating consistent detection and quantification characteristics which are required in large-scale PTM studies [5].

## 5. Enrichment Considerations

The low abundance of many of the studied PTMs limits the detection of some PTM classes by LC-MS/MS [84], so enrichment of modified peptides is needed to enhance their detection. Key to the detection of large numbers of modified peptides, affinity chromatography and immunoprecipitation techniques are used to substantially enrich PTMs and provide sufficient purification to aid the assessment of modification site-localisation by LC-MS/MS. In most cases the amount of starting material required is significantly larger than that required for expression-based proteomic studies (global proteome profiling), though efforts in the directions of using lower sample amounts have been made [90]. When combined with label and label free MS approaches, site-localisation, PTM occupancy and PTM stoichiometry for individual PTM peptidoforms can be assessed in parallel with global protein expression profiling.

Phosphorylation, glycosylation, methylation, acetylation and ubiquitination each represent distinctly different types of chemical modifications. Consequently, purification strategies have been developed to selectively enrich the different types of modified peptides prior to LC-MS/MS analysis. Although methylation, acetylation and phosphorylation are relatively small chemical modifications, they each impart significant charge-based changes to the amino acid residues they each occur on, often making detection by MS more challenging. Glycosylation and ubiquitination are larger modifications and they can introduce far more complex chemical alterations to proteins, again making MS detection challenging. The unique chemical nature of each type of PTM has led to the development of many highly specific enrichment strategies, with affinity chromatography and immuno-purification being used most often. The following section highlights some of the techniques commonly used to enrichment phosphorylated, glycosylated, methylated, acetylated and ubiquitinated proteins and the MS approaches used for site localisation and PTM quantitation.

### 5.1. Specific Enrichment Considerations

#### 5.1.1. Phosphorylation

In recent years, phosphopeptide enrichment using immobilised metal affinity chromatography (IMAC), titanium dioxide (TiO_2_) chromatography, ion exchange chromatography (SCX and SAX), hydrophilic interaction chromatography (HILIC), and immunoprecipitation (IP) have been used very successfully in large-scale phosphoproteomic quantification studies. To achieve a comprehensive assessment of phosphorylation status, larger quantities of protein are generally required compared to amounts needed for proteome only expression profiling. Following extensive refinements of the protocols for phosphopeptide purification using TiO_2_ and IMAC, enrichment of the phosphopeptide fraction can now approaches 90% in most cases, and studies can now report the identification of tens of thousands of phosphopeptides [91]. LC-MS/MS and LC-MSn approaches using CID, ECD, ETD and photodissociation have proven extremely useful for phospho-site localisation and quantification of phosphopeptides (pS, pT and pY neutral loss of HPO_3_ 79.9663 Da or H_3_PO_4_ 97.9769).

Although many of the LC/MS-based approaches used in proteomics for large-scale proteome analysis are suitable for phosphopeptide detection and quantitation, some challenges still remain. Firstly, the sub-stoichiometric phosphorylation at any given site is governed by complex biological processes beyond just the addition and remove of phosphate through kinase and phosphates activity. Secondly, the phosphate moiety is relatively labile and the actual site of the modification, and certainly the potential for quantitation, can be hampered during MS analysis. Lastly, phosphopeptides that share the same amino acid sequence will have the same precursor ion mass—this itself can impact the detection of either sequence by conventional DDA methods. For phosphopeptides that share the same amino acid sequence and precursor ion, site localisation can be challenging, particularly if multiple S, T or Y residues are in close proximity within the amino acid sequence, as the number of sequence ions capable of distinguishing individual phosphopeptides from one another reduces.

#### 5.1.2. Glycosylation

Approaches to assess protein glycosylation include the enzymatic release of *N*-glycans using N-glycosidases such as PNGase F, A or H^+^, or with various endogylycosidases such as endoglycosidase H, F1, F2, or F3, and the chemical release of *O*-glycans using reductive β-elimination under alkaline conditions. These approaches are often used to characterise the structural diversity of *N-* and *O*-linked glycans. Released glycans can be analysed in their reduced state, but quantitative analysis is complicated by the relatively labile nature of sialic acid. In such cases, derivatisation can be used to enhance quantitative analysis by MS or by incorporating chromophores to optimise quantification by UV or fluorescence detection. Glycoproteins can be enriched using either immunoaffinity and lectin affinity approaches [92], both demonstrating major advantages in glycoprotein analysis. HILIC is well suited to glycopeptide profiling, demonstrating highly specific enrichment of a broad range of glycopeptides.

Some major challenges exist for the site-specific analysis of glycopeptides by MS. The common approach involves enzymatic treatment of the glycoprotein followed by chromatographic separation and subsequent MS analysis of glycopeptides. Large glycopeptides generated using trypsin are poorly enriched using HILIC. Similarly, for MS analysis, some glycopeptides can suffer from poor ionisation efficiency, a high degree of structural heterogeneity among the different glycoforms and a lack of tandem MS spectral features to adequately characterise both the glycan and peptide backbone of the various glycoforms. In addition, identification of the site of glycosylation for N-glycans can be confounded due to the conversion of asparagine to aspartic acid following glycosidase treatment with PNGase F. The detection of aspartic acid could be used for the identification of the site of glycosylation; however, the deamidation of asparagine can result in the false identification of N-glycosites.

#### 5.1.3. Methylation and Acetylation

Early approaches assessing methylation of arginine made use of immunoprecipitation strategies and have more recently been combined with strong cation exchange and HILIC to enhance the recovery and enrichment of methylarginine containing peptides. Given the localisation of methyl groups on arginine residues, Glu-C has been used in place of the commonly used trypsin to yield additional access to complementary fragmentation data for site localisation. Recent studies have identified large numbers of methylation sites using high resolution MS [93], and by using highly specific antibodies against mono and dimethyl arginine and lysine [94].

Acetylation (monoisotopic, Ac, 42.0106 Da) of proteins has been reported at the N-terminus of proteins [95] and at lysine residues. Previous attempts to assess the “acetylome” have made use of immunoaffinity enrichment strategies, which were limited in their ability to sufficiently enrich acetylated peptides. The most in-depth proteomic studies of lysine acetylation make use of immunoprecipitation of acetylated peptides using acetyl lysine antibodies followed by further enrichment and fractionation using strong cation exchange chromatography.

#### 5.1.4. Ubiquitination

Affinity approaches using Ub specific antibodies, Ub remnant (anti diglycyl lysine antibodies), and tandem Ub binding domains have been used for enrichment of Ub proteins. The Ubisite approach, which makes us of a monoclonal antibody specific to ubiquitin and recognising remnant diglycyl lysine has demonstrated substantial enrichment of ubiquitinated proteins and was recently used to identify 63,000 ubiquitination site on 9200 proteins in two human cell lines [96].

Mass spectrometry analysis of ubiquinated peptides can result in the detection of a large number of false positives [97]. The remnant mass of ubiquitination on a target lysine residue is the diglycine sequence (Gly 75-Gly 76) (monoisotopic, 114.0429 Da). Early studies of ubiquitinated peptides on lower-resolution instruments may have resulted in false assignment of ubiquitin as leucine, isoleucine, asparagine and aspartic acid have residual masses within 1Da of the Gly–Gly remnant sequence of ubiquitin. On higher resolving instruments, asparagine (monoisotopic mass, 114.0429 Da) remains an issue when assigning ubiquitin as a PTM by tandem MS. Similarly, non-specific and artifactual alkylation of target peptides with iodoacetamide (carbamidomethylation) can result in over-alkylation (2 × 57.0215 or 114.0429 Da) of free amines at the N-terminus of peptides, or on the side chain of lysine, confound ubiquitination assignment.

#### 5.1.5. Further Considerations: Multiple PTM Studies, Studies without Enrichment

Recent examples demonstrating the combined quantification of multiple PTM types in large-scale studies provide an important stepping-stone for future PTM studies. High throughput quantitative mass spectrometry is becoming increasingly useful at mitigating the issue of sensitivity, enabling screening of several biologically relevant PTMs *en masse* and with greater ease. PTM crosstalk is outside the scope of this work, but is comprehensively discussed elsewhere [98].

Although measuring both post-translationally modified and unmodified peptides species without the need for prior enrichment appears to be achievable, current mass spectrometry technology is not currently mature enough to handle such stochastic complexity on the fly. Thus, pre-enrichment of post-translationally modified peptides is currently an inevitable step in a large-scale PTM studies. That being said, groups have attempted to circumvent this issue by leveraging the speed and sensitivity of the Orbitrap class of mass spectrometers and achieved comprehensive coverage of the commonly studied PTMs including phosphorylation and N-acetylation without specific enrichment.

## 6. Pinpointing the Modification—Site Localisation Algorithms

Peptide identification is usually the first step for bioinformatics analysis in bottom-up MS-based proteomics, and the same is true for PTM analysis. In addition to protein identification with standard search engines, unambiguous modification site localisation is an important and challenging part in PTM identification, and several specific algorithms have been proposed for automatic PTM site localisation [99,100,101,102,103].

The challenge of site-localisation stems from two main difficulties. First the database search space will expand in combinatorial fashion for each modification included, thus increasing the computation cost and false positive rate for PTM identification [12]. Second, a peptide with the same amino acid sequence can have multiple same instances of the same amino acid residues and some modifications can modify different amino acid residues within the same sequence, for example, phosphorylation can modify tyrosine (T), serine (S) and threonine (Y) residues when all of them are present in the same peptide [12,103]. Unambiguously identifying a modification site is determined by the presence of one or more fragment ions [103]. For example, a phospho-tyrosine specific immonium ion is commonly used as diagnostic information to determine the presence of a phospho-tyrosine residue [104]. However, very often these ions may be lost or not be detected by mass spectrometers [12,99].

Ascore is one of the earliest automatic site localisation algorithms for phosphorylation [103]. It is a probability-base scoring algorithm based on the presence and intensity of site-determining ions in MS/MS spectra. Site-determining ions are those fragment ions that are exclusive to a site location for a PTM peptide. Ascore is one of the most popular site location algorithms and its performance has been verified in various studies [102,105]. However, although built as a “fit-for-purpose” tool, Ascore can only work for MS/MS and CID fragmentation. With the advent of new types of peptide fragmentation techniques including HCD, ETD and ECD, the fragment ions produced are different from those of the classical CID method, and various algorithms, including Andromeda [100], PhosphoRS [105] and SLoMo [106], have been proposed to cater for these extended fragmentation methods. Mascot Delta Score, a method using the difference between the top two matches of modification sites for the same peptides in the database search, can be used for arbitrary PMT types but is limited to Mascot search results [101,102]. Most peptide search engines have now incorporated one or more PTM site localisation algorithms for PTM identification. For example, ProteinPilot (V5 Sciex) Parogon algorithm has ID focus options, which allow the additional consideration of large sets of biological post-translational modifications [107]; SEQUEST [108] uses the Ascore algorithm; MaxQuant [109] exploits the Andromeda algorithm [100]; and Mascot uses the MD score method [110].

Unlike the direct search approach used in DDA, DIA adopts a mining approach which uses a pre-built spectral library for peptide and protein identification and quantitation [111]. This approach applies to both modified and un-modified peptides. For the PTM peptides to be identified and quantified by DIA or SWATH, both the precursor ions and their fragment ions need to be included in the spectral library [112]. The quality and completeness of the spectral library is a key element to the success of PTM peptide identification. The generation of the peptide library is usually done by combining many runs of DDA experiments [111,112], using DIA pseudo spectra [113] or extending a local spectra library with one or more archived or external libraries [114,115]. Therefore, the traditional site localisation methods for DDA can also apply to DIA.

Besides these DDA-based localisation methods, some recent work has proposed methods of inferring PTM peptides directly from the spectral library [5,89]. Rosenberger et al. proposed the IPF (Inference of PeptideoForms) algorithm which can independently infer modified peptides with specific site-localization using a site-localized or unlocalized spectral library [5]. IPF uses a posterior probability estimation and Bayesian hierarchical model for site localisation. IPF has been integrated into the OpenSwath software [116]. In another similar work, Keller et al proposed a method that infers the putative PTM from the mass shifts exhibited by the precursor and missing fragment ions in the lower-ranking peak groups [89]. This functionality has been added to SWATHProphet [117]. For a more complete review on site localisation, please refer to [12,99].

There are still some limitations to the current PTM identification methods. First, the number of modifications included in each search cannot be too large due to the search space expansion, with an upper limit being about 6–10 modifications [107]. Second, though most search engines can report the estimated confidence of identification on both peptide level or dataset level using an expected score or FDR (False Discovery Rate) based on a target-decoy strategy [118], not many search engines can directly output the site assignment confidence or False Localisation Rate (FLR). Some efforts have been made with either synthetic peptides with known modification sites [102,105] or peptides with only one potential modification sites [119].

## 7. Understanding the Reproducibility of PTM Quantitation

While site localisation will enable the confident identification of modified peptides via the computational strategies described before, it is their quantification and its change with condition that is crucial to inferring relevance to disease. For this quantitative analysis to be useful, the data collected has to be of sufficient quality to enable it, and the sample size has to be adequately matched to the analysis goals. A useful discussion on sample size in the specific context of large scale PTM analyses can be found in [16]. There are many sources of variation at PTM level in proteomics—from biological, sample preparation through to LC-MS and technical instrument related [10]; these are further enhanced for PTMs, which, through their dynamic nature, can introduce increased variability.

There are two facets to quantitative variability: that of identification, such as determined for instance by the percentage of replicates in which a modification can be measured, and that of quantitation, such as measured by for instance by coefficients of variance. In terms of identification, in the case of PTMs, the overlap between replicates even for cell cultures can be quite low [16]. This low overlap will lead to a high proportion of missing values to be tackled in the subsequent analysis; the issue of missing data is of course not unique to the PTM scenario, where it stems from the stochastic aspect of peptide identification [120,121], with many methods initially developed to handle it in the context of microarrays [122] and in specific proteomics context [123]. The better resolution of current MS instruments and the increased uptake of multiplexed and DIA methods can contribute to lowering the MS variability. As demonstrated in a very recent benchmarking experiment, missing data is less of an issue with TMT though the problem accumulates when a larger number of runs is required, but more so with label free data [6].

Since the label free quantification of the PTM peptides is gaining increasing attention, several measures have been taken to overcome the missing identifications of peptides and PTM peptides. Stochastic effects caused due to sampling issues in DDA are overcome by introducing more biological replicates thus reducing the missing values. Additionally, software such as MaxQuant addresses this issue at the data processing level by performing matching between runs. Through this approach, the software transfers the identification (and thus quantitation) when there is a MS1 feature but not successful MS2 spectra for the corresponding feature. In the DIA space, a SWATH study assessing the quantification of peptidoforms [5] has been used to quantify the presence of PTMs in replicates of a large scale study of plasma from 116 twins, and finds a good median detectability of modifications in the range of 50–100 samples.

Where a large percentage of data is missing across samples, data imputation may be needed, though the validity of imputation is often a hotly debated topic among statisticians; this remains so in the case of PTM analysis. Schwammle et al. [124] recommend against PTM data imputation strategies using methods adopted from proteomics or transcriptomic studies, favouring novel analysis models that handle data absence by different statistical approaches, such as presence-absence models based on Binomial likelihood [125], combining statistical models that assess qualitative differential observation and differential expression [126] or using specific approaches tailored to large scale proteomic studies [127]. However, other established proteomic pipelines make use of data imputation adapted from microarray experiments. For instance the popular *impute* R package developed for microarray data analysis is part of well-established workflows [6].

Popular packages such as Perseus offer data imputation options including the restriction of values present in a certain percentage of samples, and allow imputing random data from a specific distribution, typically normal. The potential positive effect of matching between runs and imputation with random values strategies is well illustrated in [128]. When choosing whether or how to impute, one should carefully consider several factors: presence of replicates (as imputation will be improved if data can be consolidated across technical replicates), percentage of values imputed (if a very large proportion of the dataset ends up being imputed, then methods of imputing for sparse datasets should be well understood), data distribution after analysis, the impact of imputation (does the imputation modify the resulting data distribution), and, crucially, the stability of the obtained results under imputation. If values sampled from a random distribution are generated to fill in missing data, are the obtained results stable when a different set of random values are imputed, or will a different set of differentially expressed features be obtained for each imputation run? An evaluation of stability can be done by a bootstrapping exercise, or at least simply by repeating the imputation steps more than once and evaluating the effect on differential expression.

A second facet of reproducibility is that of quantitation, typically captured through calculating coefficients of variation or correlations among technical replicates. For clinical samples, understanding coefficients of variance for technical replicates is paramount. In a study demonstrating a new PTM enrichment method combining immunoaffinity purification and LC-MS/MS without depletion, the authors demonstrate reproducibility for plasma technical triplicates in the vicinity of 20% [69]. The large-scale aforementioned twin plasma SWATH study quantifies biological CVs for each modification, with median values in the range of 30%, reporting much lower technical and whole process variability [5]. Alternatively, reproducibility of PTM quantitation can be captured via correlations of technical or biological replicates; for instance phosphopeptide abundance correlations of over 80% were determined for biological replicates using a MaxQuant-based label-free platform [129]. In addition, at a higher level, quantitative reproducibility can be demonstrated by showing conserved processes and motifs [6], and in a more visual manner via multivariate analysis, as we discuss next.

## 8. Understanding the Changing Levels of Modified Peptides in Context 

The extraction of PTM quantitation and possible data imputation is usually followed by normalisation steps, multivariate analysis such as clustering and PCA, and differential expression of the modified peptides. These steps will typically follow the established methodology employed for general shotgun proteomic data generated using the same platform, be it labelled (metabolic or chemical), label free or DIA/SWATH. However, the increased complexity of the PTM multivariate data stems from two aspects: it is carried out at the peptide and even site level, hence yields larger datasets, and it is tightly coupled with the un-modified and protein expression level. We will first briefly discuss the multivariate analysis options, and then in more detail the relationship with protein and un-modified peptide quantitation.

A very recent review [130] describes in visual detail the downstream statistical data analysis options for shotgun proteomics in general, and highlights many of the steps can be carried out in the popular Perseus software suite [109]. Data normalisation is a necessary first step, usually carried out to remove variation stemming from uneven sample loading, with commonly employed methods including total area normalisation, median, quantile-quantile, variance stabilising normalisation, loess and many others. A systematic evaluation of normalisation methods in the context of label free proteomics using public standardised datasets can be found in [131]. The basic statistical tests used for differential expression between conditions remain the standard approaches such as ANOVA or t-tests for differential expression, or their variants such as moderated t-tests accounting for variance shrinkage across the whole dataset [132]. Developed initially in the context of microarrays, these methods are available in the popular R packages *limma* [133] and SAM [134], with the latter being used in the phosphoproteomics specific context [6].

Because PTMs are quantified at the peptide rather than protein level, the scale of the resulting datasets will be much larger, hence approaches to limit false discoveries when doing repeated tests must be considered. Most commonly used methods include Benjamini and Hochberg (BH) corrections [135], Storey and Tibshirani q-values [136] or more stringent cut-offs if deemed appropriate. For instance, the TMT protocol [8] employed Student *t*-test BH-corrected *p*-values 0.001 in addition to fold change requirements of 1.75.

Importantly, a change in abundance in a PTM peptide across conditions of interest could either reflect a change in post translation modification patterns, or a change in abundance of the protein itself [137], thus determining protein abundance changes side-by-side with the PTM changes is crucial. This was demonstrated in a seminal phosphorylation study [138] showing that 25% of the changes in differentially expressed phosphopeptides could be attributed to protein expression changes, thus underscoring the need to account for protein amount normalisation, or at any rate side by side comparison of protein and PTM ratios. However, the process of specific PTM enrichment (if carried out) uncouples the PTM abundance from the protein amount [12], potentially making such normalisation difficult unless the enrichment flow-through is retained.

The stoichiometry—also known as site occupancy—represents the fraction of modified peptides as a percentage of the total protein amount, and provides insight in the down-stream analysis, as a high occupancy coupled with differential regulation of the modification can be a good indicator that the modification is functional [84]. Determining it is a novel part of the PTM data analysis, one not covered by the standard methods for multivariate analysis described above, and one that can be particularly challenging for modifications like phosphorylation, which are both occurring on a rapid time scale, and at low abundance. Stoichiometry is difficult to determine in a standard mass spectrometry PTM analysis, due to the different behaviour of modified and un-modified peptides which makes it hard to compare them directly [139]. If the relative change in protein amounts is captured alongside the relative change in modified peptides, then the relative change in stoichiometry can be understood; however the absolute occupancy will not be directly known [140]. A workflow for determining absolute phosphorylation stoichiometry using stable isotope labelling on a proteome-wide scale has been described in [140], and further improvements have been made to it by using isobaric labelling in a 10-plex TMT setup [141]. In the label-free scenario, Sharma et al describe a computational strategy for determining fractional occupancy of phosphorylated peptides using MaxQuant [129].

## 9. Online Tools for Subsequent Analysis

**Figure 3:**
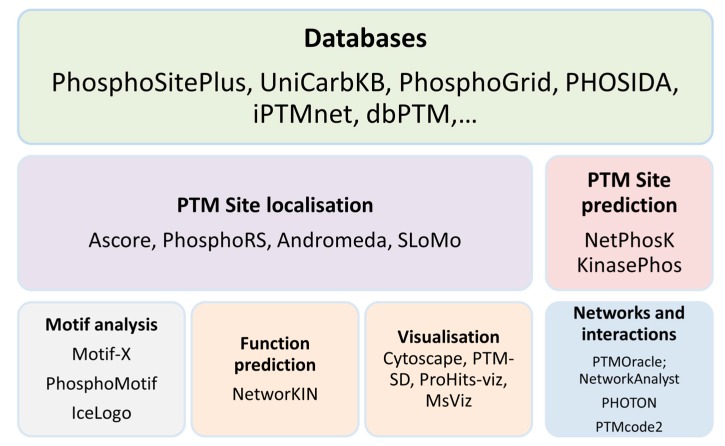
PTM tool categories, and a few highlighted examples.

Once PTMs are identified and quantitated, there are numerous tools available for subsequent analysis, and of course numerous databases underpinning the analysis and making prediction possible; PTM databases have been previously reviewed in depth [142]. There are a variety of broad tool categories (databases, tools dealing with site localisation and prediction, motif analysis, function prediction and interaction—Figure 3 captures the main tool categories), and below we highlight some of the more recent additions (Table 2 and Table 3). In addition, due to the more complex challenges of glycan analysis, a vast array of glycoproteomics specific informatics tools have been developed—a recent in-detail book chapter surveys databases and tools in the specific glycomics context [143].

Many public databases are available for searching for PTM annotation, some are type-specific such as DEPOD [144] and Phospho.ELM [145] and others cover more modification types such as Uniprot [146], PHOSIDA [147], PTMcode [148]. Some only include experimentally verified PTMs such as Phospho.ELM, PhosphoSitePlus [149], PhosphoGrid [150] and UniCarbKB [151], some include known and predicted functional annotations between PTMs such as iPTMnet [152]. Refer to [153,154] for good reviews of PTM databases and the modification type coverage. Building on from the available annotation, PTM site prediction tools leverage the available information in PTM databases, and machine learning algorithms, to predict new PTM sites and binding motifs.

Motif analysis can provide useful sequence pattern information for all identified PTM proteins, and helps identify the significant motifs which, in turn, based on known substrate specificity, can ideally help identify the enzymes involved—though the latter part is still extremely challenging. Motif-x is arguably the most popular tool for identifying the significant motifs in existing PTM peptides [155]. Many studies have used Motif-x in their PTM analysis as a validation of existing motifs or discovery of novel motifs [9,156,157].

Many of the existing PTM analysis tools offer great data visualisation features. For example, Cytoscape with its many visualization apps including PTMOracle and STRING for PPI and networks; Protein data bank and PTM-SD for protein structures [158]; ProHits-viz [159], a suite of web tools for visualizing interaction proteomics data; MsViz [160], a graphical tool for manual validation and quantification of PTMs for small to medium scale experiments.

## 10. Methods of Functional Analysis

**Figure 4:**
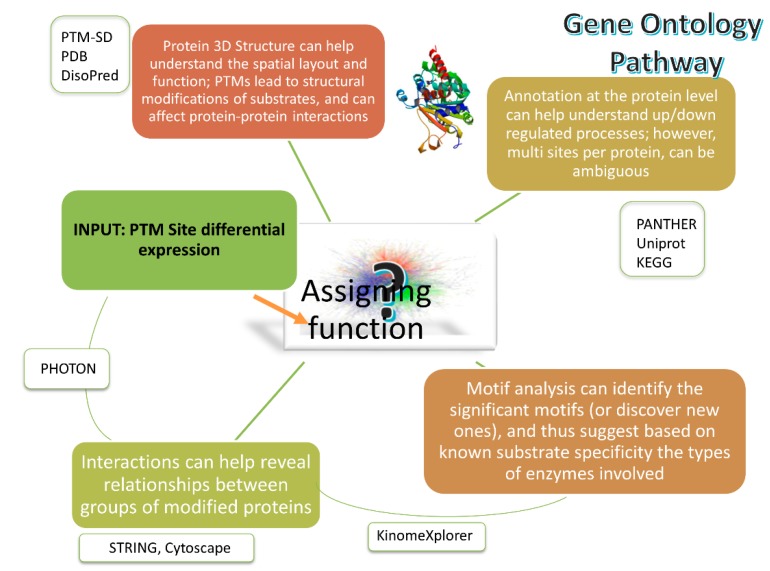
Areas relevant to the task of predicting functions of PTM modifications. Several integrative approaches are emerging. Orange arrow indicates the input to the functional analysis tools; green lines indicate the various aspects of PTM functional analysis.

A PTM can either modify protein structures, regulate functions or add a new group [11]. After the quantification analysis of PTM data, and accessing available databases to check whether the PTM/motif is known, we need to understand the complex circuitry of cell signal transmission that the PTM impacts. Functional analysis aims to bring the quantitative PTM analysis into biological contexts based on annotations, protein structures, protein interactions, pathways and networks and interpret the data at the biology level (Figure 4). In this section, we review the general methods currently available for PTM functional analysis.

Using the annotations in publicly available databases can help PTM functional analysis from various aspects. Firstly, using ontology categories, such as GeneOntology (GO) and PhosphoSite ontology, can help classify and understand the identified PTMs from the functional levels. For example, the GO molecular function and biological process categories retrieved from PANTHER [178] were used to compare phosphoproteins and total proteins in porcine muscle [157]. A distribution of phosphoprotein types was generated by using the PhosphoSite ontology to classify phosphoproteins of lung cancer cell lines and tumors [23].

Post translational modifications yield structural changes in the substrate, which in turn can affect function and protein-protein interactions—hence 3D structure analysis is important for understanding functional relationships of PTM proteins. The Protein Data Bank [179] is an extensive publicly available data repository of protein 3D structures. PTM-SD [158] provides structurally resolved and experimentally annotated PTMs in protein structures in 3D views. Sharman et al. used DisoPred software [180] to predict disorder signal state for all proteins and mapped to phosphorylated sites and found that protein phosphorylation tends to occur to disordered regions in stimulated cells while independent of structures in unstimulated cells [129].

Protein-protein interaction (PPI), network and pathway analysis leverage PTM functional analysis from single-protein-based to group-of-proteins-based. There are many tools available for protein interaction studies, and some of the most popular ones are listed in Table 4. The group of proteins is usually a set of proteins of biological interest, such as the set of proteins showing a common regulation trend from the quantification analysis. By putting these proteins in the pathway or network context, protein lists are mapped to biological pathways or PPI networks which aids the interpretations and visualisation. There are several tools available for pathway and network analysis for general proteomics data and they vary in the use of function and topological information [181]. However, pathways and networks analysis tools specific for PTMs are still very limited [182]. Pathway and network analysis can offer insight into PTM functions and discover novel pathways or networks; for example, important PPI networks were discovered and different regulatory metabolism mechanisms were revealed for phosphoproteins in porcine muscle proteins by using STRING [9,157]. PTM crosstalk analysis, which aims to identify relationships between different types of PTMs, can also be aided by PPI networks. For example, Grimes et al. integrated three types of PTMs, i.e., phosphorylation, methylation and acetylation, in lung cancer cell lines and outlined lung cancer cell signalling networks [183].

Recent studies combine several of these approaches; for instance, the workflow in [156] starts from the extraction of crosstalk motifs for human PTM peptides downloaded from PhosphoSitePlus using Motif-x and then goes through gene ontology enrichment analysis, kinase analysis with NetworkKIN [184] and network analysis using Cytoscape with the GeneMANIA app [185].

For phosphorylated proteins, kinase analysis can reveal the relationships between phosphorylation sites and the protein kinases. NetworkKIN is a popular tool to model phosphorylation networks by using Kinase-substrate relationships. Qi et al. re-constructed kinase-substrate phosphorylation networks by using predicted site-specific kinase-substrate relation for mouse testis [186]. Rikova et al. characterized tyrosine kinase signalling across non-small cell lung cancer cell lines and tumors and identified known and novel oncogenic kinases [23]. In addition, kinase-substrate enrichment analysis can be used to performed enrichment analysis for phosphorylated substrate groups [187].

Recent integrative approaches have combined several of the steps outlined above, in order to help get closer to extracting meaningful information rather than just providing long lists of PTM sites. The PHOTON tool [188] takes as inputs, sets of differentially quantitated PTMs as arising from quantitative workflows reviewed here, and protein-protein interaction data, and through network-based statistical modeling, generates scores to identify significantly functional signalling proteins. Other developing approaches include modified versions of the single sample gene set enrichment analysis approach [189] tailored to the PTM specific context (PTM-SEA making use of PTMsigDB database). Such tools, when mature and in common use, will speed up the latter, difficult part of PTM functional analysis.

## 11. Concluding Remarks

The interest in post translational modifications is growing and will continue to be sustained, due not just to increased understanding, but to the practical importance and likely payoff for such studies in the clinic. As quantitative techniques such as TMT and SWATH mature, they will yield increased information in the databases and hence better capacity for the predictive tools, which continue to develop. While keeping in mind the caveats about correct site assignments and some of the other MS related challenges remaining, arguably the part of the workflow ending with generating relative quantitation and relative stoichiometries is getting increasingly reliable and routine. Analysing several PTMs at the same time and PTM crosstalk remains difficult, and for many modifications there is little available data and few usable tools. Function prediction remains a difficult step, though we have seen the beginning of computational tools blending protein interaction data with PTM abundance ratios to generate functional predictions, which constitutes a big step forward. However, validation of the results will still be key, at many levels, from the predicted site assignments, predictive tools performance and all the way through to the predicted enzymes activity.

## Figures and Tables

**Table 1:** Examples of PTM studies for various clinically relevant biological fluids.

Body Fluid	PTM	Disease	Description	Reference
Plasma	Phosphorylation	Breast cancer	Proteomics study revealed unique phosphopeptide profile of plasma derived extracellular vesicles from breast cancer patients	[60]
	Phosphorylation	Alzheimer’s disease	Phospho-Tau presence in AD patient plasma samples	[61]
	Phosphorylation	Parkinson’s disease	Plasma samples demonstrated higher phosphorylated α-synuclein levels compared to healthy controls	[62]
	Acetylation, Ubiquitination	Glioblastoma	LC-MS/MS analysis on plasma from patients with glioblastoma multiform showed decreased acetylated and ubiquitinated peptides	[63]
	Ubiquitination	Leukaemia	Unique plasma profile of ubiquitin-proteasome system (UPS) and steep ubiquitin protein levels were detected in AML and ALL patient plasma samples	[64]
Sera	Glycosylation	Alzheimer’s disease	Increased O-GlcNAcylation levels and decreased global glycosylation levels	[65]
	Glycosylation	Hepatocellular Carcinoma	Increase in the levels of golgi glycoprotein GP73 in HCC patient serum	[66]
	Glycosylation	Ovarian cancer	Changes in serum glycome profile of ovarian cancer patients	[67]
	Glycosylation	Breast cancer	Abundant fucosylation in metastatic breast cancer patient sera	[68]
	Acetylation, Methylation	Leukaemia	Lysine acetylation and arginine mono-methylation as the prevalent PTMs in sera of patients with acute myelogenous leukemia, breast cancer, and non-small-cell lung cancer	[69]
Saliva	Phosphorylation	Control	Large-Scale Phosphoproteomics Analysis of Whole Saliva	[70,71]
	Glycosylation	Oral ulcer	Proteomic and N-glycoproteomic quantification reveal aberrant changes in the human saliva of oral ulcer patients.	[72]
	Glycosylation	Control	Analysis of age and gender associated N-glycoproteome in human whole saliva	[73]
	Glycosylation	Control	Identification of N-Linked Glycoproteins in Human Saliva	[74]
Cerebro spinal fluid	Glycosylation	Alzheimer’s disease	Unusually glycosylated acetylcholinesterase in CSF samples of AD patients as a diagnostic molecule	[30]
	Glycosylation	Schizophrenia	Identification of N-glycosylation changes in the CSF and serum in patients with schizophrenia	[75]
	Phosphorylation	Alzheimer’s disease and Parkinson’s disease	CSF of AD and PD patients have also been positive for phospho-Tau and phospho α-Synuclein respectively	[20,76]
	Ubiquitination	Alzheimer’s disease and Parkinson’s disease	Accumulation of other PTM, ubiquitin and associated enzymes in the CSF samples of AD and PD patients	[77,78]
Pancreatic fluid	Several	Pancreatitis	Use of pancreatic fluid to outline the PTM profile unique for individuals with chronic pancreatitis	[79]
Urine	Glycosylation	Prostate cancer	Characterisation of Glycoproteins from urine samples of prostate cancer patients with different Gleason scores	[80]
	Glycosylation	Prostate cancer	Investigation of glycoproteome to discriminate prostate cancer (PCa) from benign prostatic hyperplasia (BPH)	[81]
	Phosphorylation	Pregnancy	Evaluating the expression of specific phosphoproteins during pregnancy comparison with non-pregnancy.	[82]
	Phosphorylation	Bladder cancer	Phosphorylation of urinary tyrosine protein reported for predicting early bladder cancer onset	[83]

**Table 2:** PTM annotation databases examples.

Name	Description	Accessibility	Reference
DEPOD	Manually curated database of human active phosphatases	http://www.depod.bioss.uni-freiburg.de/	[144]
Phospho.ELM	Relational database of in vivo and in vitro phosphorylation data	http://phospho.elm.eu.org/	[145]
UniProt-GOA	Database of gene ontology annotations to UniProt Proteins	https://www.ebi.ac.uk/GOA	[161]
PHOSIDA	Database of phosphorylation data from in-house proteomics studies	https://www.biochem.mpg.de/1144243/Phosida http://www.phosida.de/	[162]
PhosphoSitePlus	Manually curated resource of experimentally determined Human and Mouse PTMs	https://www.phosphosite.org/homeAction.action	[163]
PhosphoGrid	Database of experimentally determined PTM sites in Saccharomyces cerevisiae	https://phosphogrid.org/	[150]
UniCarbKB	Curated knowledgebase for glycomics and glycobiology research	http://www.unicarbkb.org/	[151,164]
iPTMnet	An integrated database of PTMs in systems biology proteins	https://research.bioinformatics.udel.edu/iptmnet/	[152]
PoGo	Mapping of peptides with PTMs and quantitation to reference genome annotation.	https://www.sanger.ac.uk/science/tools/pogo	[165]
PhosPhAt 4.0	Database and predictor of Arabidopsis thaliana phosphorylation sites based on mass spectrometry experiments.	http://phosphat.uni-hohenheim.de/	[166]
P3DB (Plant Protein Phosphorylation DataBase)	An integrated resource of proteins phosphorylation sites for different plants	http://www.p3db.org/	[167,168]
Human Proteinpedia (Human Protein Reference Database)	An integrated resource of proteins annotations including PTMs derived from different experimental techniques	http://www.humanproteinpedia.org/	[169]
dbPTM	An integrated database of experimentally verified phosphorylation sites encompassing structural and functional analysis along with disease associations	http://dbptm.mbc.nctu.edu.tw/	[170]

**Table 3:** Examples of recent MS-specific PTM related tools.

Name	Description	Accessibility	Reference
Specialize (Spectra of complex-PTModified peptides identification tool)	A tool to identify peptides and proteins with PTMs from MS spectra	http://proteomics.ucsd.edu/softwaretools/specialize/	[171]
Protein Prospector	A set of tools to detect and identify the PTMs in through searching for mass shifts in MS spectra	http://prospector.ucsf.edu/prospector/mshome.htm	[172]
Byologic, Byonic, Intact Mass, Byomap (Glyco Analysis)	A set of tools to detect and identify N- and O- linked glycans in peptides gathered through MS	https://www.proteinmetrics.com/workflows/	[173]
ScaffoldPTM (ProteomeSoftware)	A tool based on Ascore algorithm to evaluate the assignment of PTM sites on MS-based identified peptides	http://www.proteomesoftware.com/products/ptm/	[103,174]
PhoshoPep 2.0	Set of tools to study proteins pathways and interactions for MS-derived phosphorylation data from Drosophila melanogaster, Homo sapiens, Caenorhabditis elegans and Saccharomyces cerevisiae.	http://www.unipep.org/phosphopep/index.php	[175]
ProteomeScout	An integrated resource of proteins PTMs, experimental data, analysis suite and visualizations.	https://proteomescout.wustl.edu/	[176]
MsViz	An interactive software for manual validation and relative quantitation of PTMs acquired through MS experiments along with spectra visualizations.	http://msviz-public.vital-it.ch/#/about	[160]
ProSight Lite	A software tool to align a protein sequence and its PTMs and glycosylation site against MS spectra	http://prosightlite.northwestern.edu/	[177]

**Table 4:** Tools for protein interaction analyses that can also be useful in PTM context.

Name	Description	Accessibility	Reference
**STRING**	A database of known and predicted protein–protein interactions from experimental and knowledgebase sources	https://string-db.org/	[190]
**NetworkKIN**	An approach for motif-based predictions of kinases and phosphoproteins	http://networkin.info/	[184]
**Cytoscape**	An open source software for molecular interaction networks integration, analysis and visualization, along with gene annotations	https://cytoscape.org/	[191]
**ProHits-viz**	A suit of tools to perform analysis and visualization of quantitative protein interaction data	https://prohits-viz.lunenfeld.ca/	[159]
**PTMOracle**	A Cytoscape application for co-visualization and co-analysis of PTMs and protein-protein interactions	http://apps.cytoscape.org/apps/ptmoracle	[192]
**NetworkAnalyst**	An integrated tool to perform gene annotations and protein-protein interaction network analysis along with visualizations	http://www.networkanalyst.ca/	[193]
